# Disulfidptosis-associated lncRNAs predict breast cancer subtypes

**DOI:** 10.1038/s41598-023-43414-1

**Published:** 2023-09-27

**Authors:** Qing Xia, Qibin Yan, Zehua Wang, Qinyuan Huang, Xinying Zheng, Jinze Shen, Lihua Du, Hanbing Li, Shiwei Duan

**Affiliations:** 1 Key Laboratory of Novel Targets and Drug Study for Neural Repair of Zhejiang Province, School of Medicine, Hangzhou City University, Hangzhou, 310015 Zhejiang China; 2 College of Pharmacy, Zhejiang University of Technology, Hangzhou, 310014 Zhejiang China

**Keywords:** Breast cancer, Cancer genomics, Epigenetics

## Abstract

Disulfidptosis is a newly discovered mode of cell death. However, its relationship with breast cancer subtypes remains unclear. In this study, we aimed to construct a disulfidptosis-associated breast cancer subtype prediction model. We obtained 19 disulfidptosis-related genes from published articles and performed correlation analysis with lncRNAs differentially expressed in breast cancer. We then used the random forest algorithm to select important lncRNAs and establish a breast cancer subtype prediction model. We identified 132 lncRNAs significantly associated with disulfidptosis (FDR < 0.01, |R|> 0.15) and selected the first four important lncRNAs to build a prediction model (training set AUC = 0.992). The model accurately predicted breast cancer subtypes (test set AUC = 0.842). Among the key lncRNAs, LINC02188 had the highest expression in the Basal subtype, while LINC01488 and GATA3-AS1 had the lowest expression in Basal. In the Her2 subtype, LINC00511 had the highest expression level compared to other key lncRNAs. GATA3-AS1 had the highest expression in LumA and LumB subtypes, while LINC00511 had the lowest expression in these subtypes. In the Normal subtype, GATA3-AS1 had the highest expression level compared to other key lncRNAs. Our study also found that key lncRNAs were closely related to RNA methylation modification and angiogenesis (FDR < 0.05, |R|> 0.1), as well as immune infiltrating cells (*P*.adj < 0.01, |R|> 0.1). Our random forest model based on disulfidptosis-related lncRNAs can accurately predict breast cancer subtypes and provide a new direction for research on clinical therapeutic targets for breast cancer.

## Introduction

Breast cancer is one of the most common malignancies in women and is responsible for the highest mortality rate among women^1^. It is a genetically and clinically heterogeneous disease with multiple subtypes that have distinct molecular features^2^. PAM50 technology can detect the expression levels of 55 genes and divide breast cancer into 5 subtypes: Luminal A (LumA), Luminal B (LumB), HER2-enriched (Her2), Basal-like (Basal), and Normal-like (Normal)^3,4^. However, PAM50 assay is expensive and difficult to perform^5^, necessitating the need for new, less expensive alternatives to predict breast cancer subtypes.

Disulfidptosis is a newly discovered form of cell death^6^. Under glucose-deficient conditions, cells with high expression of SLC7A11 consume large quantities of NADPH, leading to an abnormal accumulation of disulfides such as cystine. This results in disulfide stress and rapid cell death^7^. It has been found that levels of methionine and cysteine are increased in colorectal cancer tissues^8^. Hydrogen sulfide (H2S) and related reactive sulfur species (RSS) help cancer cells adapt to the immune microenvironment^9^.

Long noncoding RNAs (lncRNAs) are transcripts longer than 200 nucleotides that do not encode proteins^10^. lncRNAs can act as decoys, scaffolds, and enhancers, and are involved in chromatin remodeling and transcriptional and post-transcriptional regulation^11^. There is accumulating evidence that lncRNAs often play oncogenic or tumor suppressor roles in human cancers^12,13^.

Machine learning (ML) is a powerful data analysis technique^14^ that leverages algorithms capable of processing complex functions to construct highly accurate predictive models. ML finds applications across various domains of clinical research, enabling breakthroughs such as the detection of COVID-19^15^, the diagnosis of coronary artery disease^16^, the identification of prostate cancer^17^, and the classification of leukemia subtypes^18^. One noteworthy ML algorithm is Random Forest (RF), which belongs to the ensemble learning category. RF harnesses the collective power of numerous individual decision trees for tasks like classification and feature selection^19^. In this collaborative process, each tree within the random forest makes predictions and casts votes, with the class garnering the most votes ultimately becoming the prediction of the overall model^20^. The distinguishing advantage of the RF model lies in its teamwork approach, akin to having a team of classifiers. These individual “team members” work synergistically to derive the final prediction result, delivering remarkable efficiency and exceptional accuracy^21^. Remarkably, despite the extensive exploration of machine learning techniques in the context of breast cancer prediction^22^, no prior studies have delved into the utilization of Random Forest models for predicting breast cancer subtypes. By integrating the strengths of the RF algorithm into the realm of breast cancer subtype prediction, we aim to unlock new avenues of insight and potentially enhance the accuracy of this critical healthcare application.

## Materials and methods

### Datasets

Data collection and processing were carried out as follows. Given the TCGA database’s comprehensive amalgamation of genetic, clinical, and image data spanning diverse tumor types, it stands as an indispensable asset in the field of cancer research. Therefore, our initial step involved procuring RNA-seq data for breast cancer and adjacent normal tissues directly from the TCGA database (https://portal.gdc.cancer.gov/). Subtype classification of TCGA-BRCA patients was then obtained (Table S1)^23^. After excluding patients with unknown subtypes, a total of 1104 tumor samples and 113 adjacent normal samples remained. Genes with zero expression in more than half of the patients were removed, resulting in the extraction of 3305 lncRNAs. The 1104 patients were randomly divided into a training group (776 patients) and a test group (328 patients) using the caret package in R statistical software.

### Identification of differential lncRNAs associated with disulfidptosis

The voom algorithm of the limma package (version 3.52.4)^24^ in R software (version 4.3.0) was used to identify lncRNAs that were differentially expressed between breast cancer tissues and adjacent normal tissues. lncRNAs were considered significantly differentially expressed if they had a false discovery rate (FDR) of less than 0.05 and an absolute log2 fold change (|logFC|) of greater than or equal to 1. The relationship between differentially expressed lncRNAs and disulfidptosis-related genes was then assessed using the Pearson correlation score. A significant correlation was defined as having an absolute correlation coefficient (|R|) greater than 0.15 and an FDR less than 0.05.

### Establishment and evaluation of breast cancer subtype prediction model

The Random Forest (RF) algorithm in the Random Forest R software package (version 4.7–1.1)^25^ was used for gene selection and model building by reducing the feature dimension based on variable importance (VIMP) and minimum depth. The article explains several key performance metrics for classifier assessment. AUC, denoting the area under the ROC curve, serves as a crucial gauge for classifier performance. Specificity (Sp) quantifies the proportion of accurately classified negative samples, while Sensitivity (Sn) measures the proportion of correct classifications among actual positive samples. Precision, on the other hand, estimates the ratio of correctly classified positive samples within the overall positives. The F1 score offers valuable insights into classification balance. To assess the breast cancer subtype prediction model effectively, employ a comprehensive evaluation comprising AUC, Sp, Sn, Precision, and F1 score. The SHAP package^26^ in Python was used to provide the degree of influence of each feature in the model and its positive or negative impact on each predicted outcome for explaining machine learning models.

### Immune infiltration analysis based on disulfidptosis-associated lncRNAs

Immune infiltration was analyzed using the CIBERSORT deconvolution algorithm^27^ on R software to calculate the composition of tumor immune cells from expression profiles. Single-Sample Gene Set Enrichment Analysis (ssGSEA) in the GSVA R software package (version :1.44.5)^28^ was used to calculate the degree of infiltration of 28 immune cell types based on published immune cell gene signatures^29^. The correlation between disulfidptosis-related lncRNAs and immune infiltration was calculated to explore their relationship in different breast cancer subtypes.

### Interaction of RNA methylation with disulfidptosis-associated lncRNAs

RNA methylation modifications are key regulators that affect cellular biological functions such as cell proliferation and metastasis, stem cell differentiation, and homeostasis in cancer^30^. Three RNA methylation modification-related genes were obtained from the literature, including 23 m6A modification genes^31^, 12 m5C modification genes^32^, and 10 m1A modification genes^33^. Correlations between disulfidptosis-associated lncRNAs and RNA methylation genes were calculated to explore their relationship in different breast cancer subtypes.

### Interaction of angiogenesis with disulfidptosis-associated lncRNAs

Angiogenesis, the process of forming new blood vessels from pre-existing vessels, is an important event in tumor growth and hematogenous metastasis^34^. A set of 36 angiogenesis-related mRNAs was obtained from the Hallmark gene set^35^. The association between disulfidptosis-associated lncRNAs and angiogenesis-associated genes (AAGs) was assessed to explore their relationship in different breast cancer subtypes.

### Statistical analysis

Statistical analyses were performed using R version 4.1.0. Gene expression in tumor tissue was compared to that in adjacent non-tumor tissue using a t-test. Correlations between genes were calculated using Pearson analysis, and differences in proportions between groups were compared using Wilcoxon tests. The corrected *P* value (*P*.adj) was calculated using the Bonferroni method. The performance of the models was evaluated using receiver operating characteristic (ROC) curves and the area under the curve (AUC). Two-sided tests were used to report *p*-values, with values less than 0.05 considered statistically significant.

### Ethics approval and consent to participate

TCGA belong to public databases. The patients involved in the database have obtained ethical approval. Users can download relevant data for free for research and publish relevant articles. Our study is based on open-source data, so there are no ethical issues and other conflicts of interest.

## Results

### Enrichment analysis of disulfidptosis-related genes

**Figure 1:**
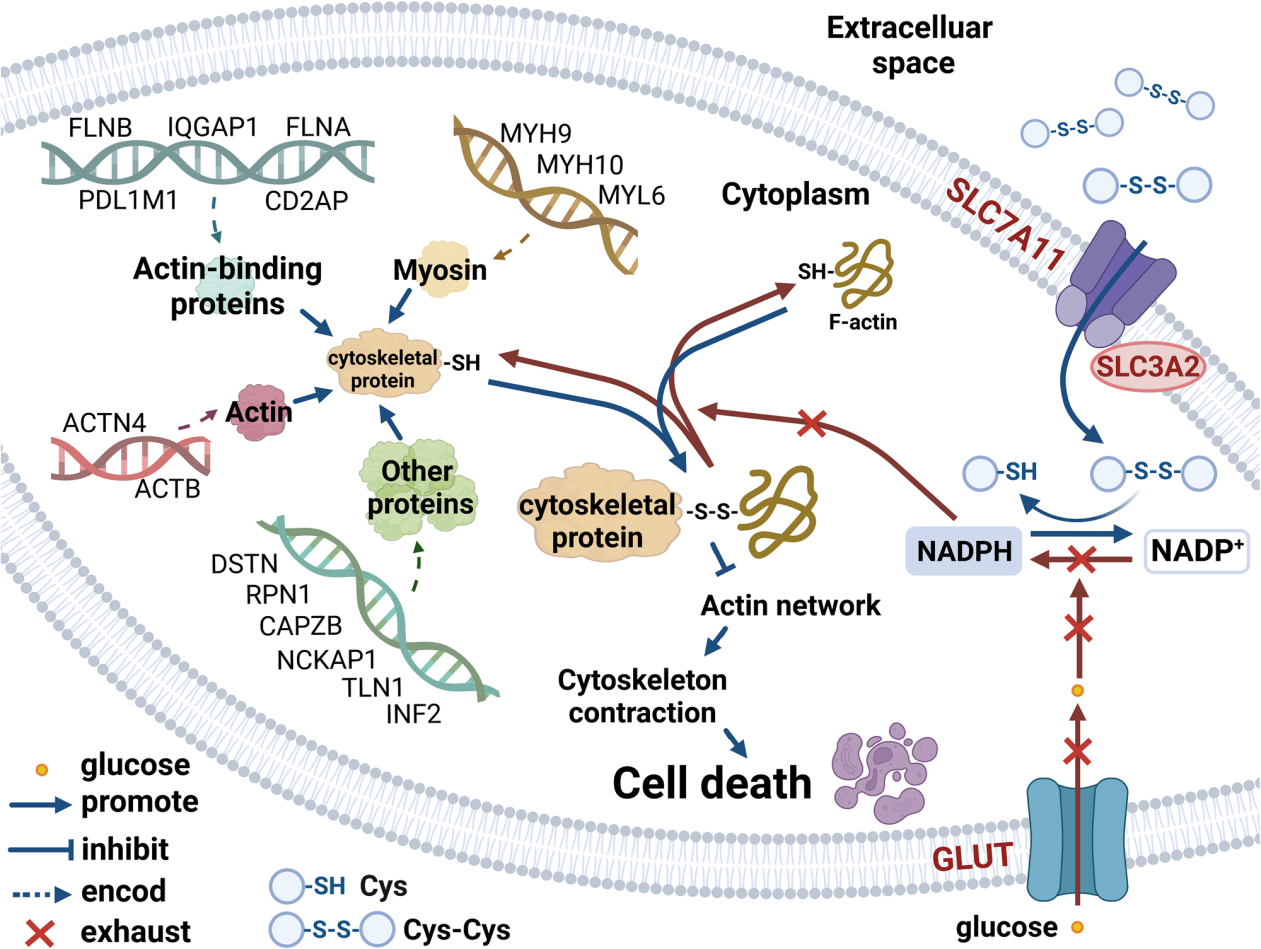
Molecular mechanism of disulfidptosis-related genes. Under glucose-deficient conditions, high expression of SLC7A11 mediates cystine uptake into cells, consuming a large amount of NADPH and reducing cystine to cysteine, resulting in NADPH depletion. This promotes the oxidation of the sulfhydryl group (–SH) on the actin cytoskeleton protein’ s cysteine to form intermolecular or intramolecular disulfide bonds (–S–S–), leading to cytoskeleton collapse and plasma membrane separation, eventually inducing cell death. SLC3A2 encodes a chaperone protein for SLC7A11. NADPH promotes cytoskeleton reorganization by regulating actin polymerization and depolymerization and plays an important role in maintaining cytoskeleton stability and plasticity. INF2, DSTN, TLN1, CAPZB, RPN1, and NCKAP1 are involved in actin synthesis and cytoskeleton formation. FLNA, FLNB, IQGAP1, PDLIM1, and CD2AP are actin-binding proteins that regulate protein function by binding to actin. MYL6, MYH9, and MYH10 are myosin proteins that interact with actin to form part of the cytoskeleton. ACTN4 and ACTB are intracellular actins that maintain cytoskeleton stability. (Created by BioRender, https://www.biorender.com/).

We conducted an enrichment analysis of disulfidptosis-related genes by identifying 19 disulfidptosis-related mRNAs through a literature search (Fig. 1). Under glucose-deficient conditions, high expression of SLC7A11 mediates cystine uptake into cells, consuming large amounts of NADPH and reducing cystine to cysteine, resulting in NADPH depletion. This promotes the oxidation of the sulfhydryl group (–SH) of cysteine on actin cytoskeletal proteins to form intermolecular or intramolecular disulfide bonds (–S–S–), leading to the collapse of the cytoskeleton and separation of the plasma membrane, eventually inducing cell death^6,7,36^. INF2 and PDLIM1 are involved in actin synthesis and have the unique ability to accelerate actin polymerization and depolymerization^37^. CD2AP can recruit capping proteins to specific subcellular locations and modulate their actin capping activity through allosteric effects to affect actin assembly^38^. MYH9 and MYH10 interact with actin to become part of the cytoskeleton. Under glucose-deficient conditions, disulfide bonds can form between MYH9 and MYH10 proteins, leading to abnormal protein function^39,40^. ACTN4, FLNA, FLNB, IQGAP1, and TLN1 are intracellular actin-binding proteins that maintain cytoskeleton stability^41–43^. MYL6 is involved in muscle contraction and cell motility by interacting with actin and myosin heavy chains^44^. Aberrant expression and aggregation of ACTB can affect cytoskeletal changes^45^. DSTN promotes depolymerization and reorganization of actin and is involved in cytoskeletal remodeling and regulation of actin filament turnover^46^. CAPZB plays an important role in regulating the dynamics of actin filaments and stabilizing their length^47^. RPN1 and NCKAP1 are cytoskeletal proteins involved in upregulating Arp2/3 complex-mediated actin nucleation^48^.

### Feature selection

**Figure 2:**
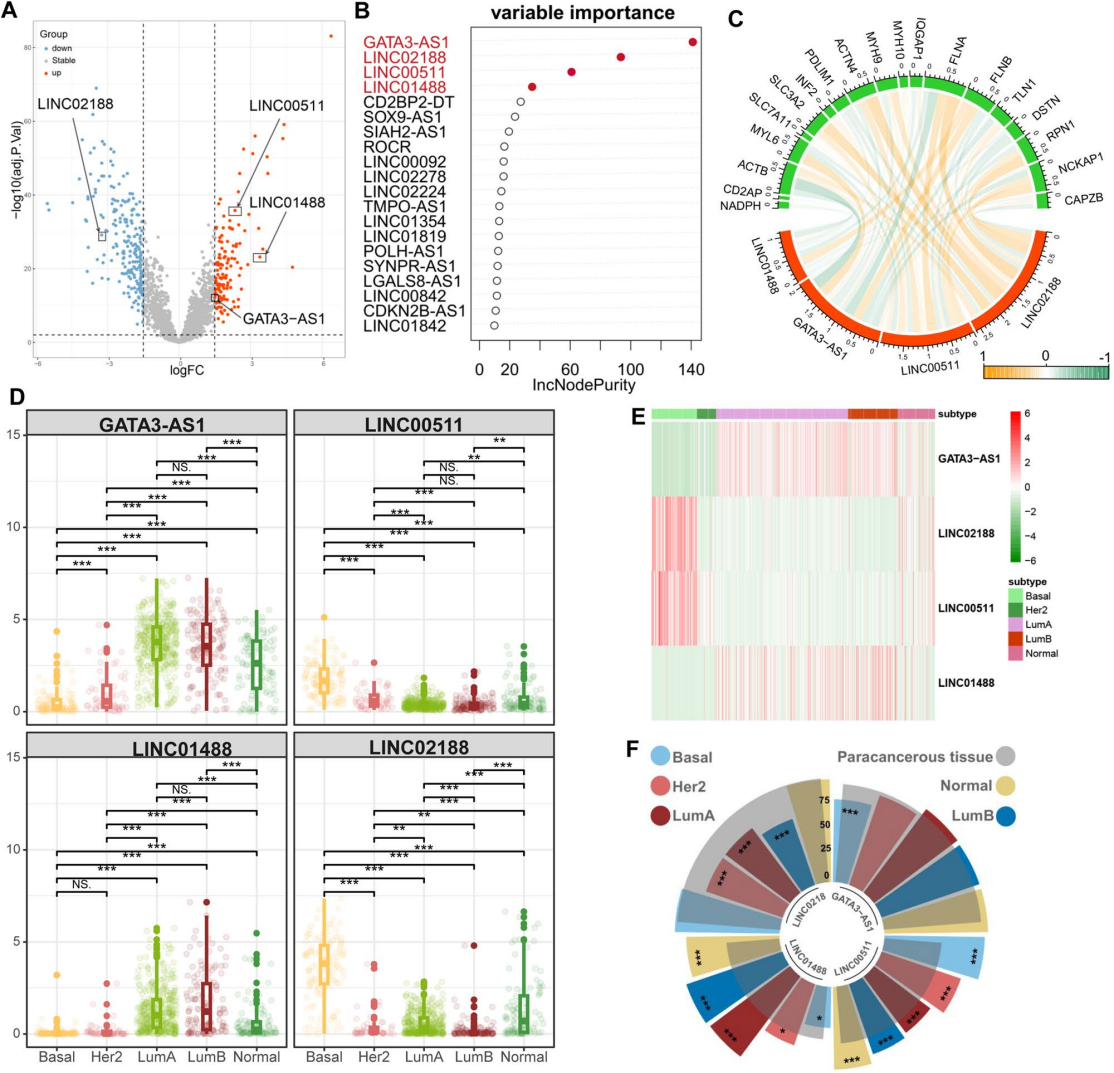
Expression of key lncRNAs in breast cancer subtypes. (**A**) key lncRNAs are differentially expressed between breast cancer and paracancerous cells. Red indicates up-regulated genes while blue indicates down-regulated genes. Volcano Plot was constructed through “ggplot2” R package (version:3.4.2, https://cran.r-project.org/web/packages/ggplot2/index.html). (**B**) Four genes with relative importance above 40 are selected from the top 20 features to build a model. (**C**) Disulfidptosis-related genes show significant correlation with key lncRNAs. The thickness of the connecting lines in the figure represents the value of Pearson’s R. Correlation Chord Diagram was constructed through “circlize” R package (version:1.0.12, https://cran.r-project.org/web/packages/circlize/index.html). (**D**) The box plot displays the significant differential expression of key lncRNAs across different breast cancer subtypes. * indicates *P* < 0.01, ** indicates *P* < 0.001 and *** indicates *P* < 0.0001. Boxplot was constructed through “ggplot2” R package. (**E**) A heatmap shows the expression of key lncRNAs in different subtypes. Heatmap was constructed through “pheatmap” R package (version:1.0.12, https://cran.r-project.org/web/packages/pheatmap/index.html). (**F**) The height of the bars represents the expression quantile of key lncRNAs. we calculated the quantile ranks of key lncRNAs among all non-zero expressed lncRNAs in the four subtypes of breast cancer. LINC00511 had high abundance in the five subtypes (0.75–1.0 quantile, Q4); LINC00511 has a low proportion in paracancerous tissues (0.25–0.5 quantile, Q1). GATA3-AS1 is highly abundant in all five subtypes and their paracancerous tissues (0.75–1.0 quantile, Q4). LINC01488 has a low abundance in the Basal subtype (0.25–0.5 quantile, Q2). LINC02188 has a high abundance in the Basal, Her2, LumA, Normal subtypes and paracancerous tissue (0.75–1 quantile, Q4). *Indicates *P* < 0.01, ** indicates *P* < 0.001 and *** indicates *P* < 0.0001. Circular barplot was constructed through “tidyverse” R package (version:2.0.0, https://cran.r-project.org/web/packages/tidyverse/index.html).

A total of 345 differentially expressed lncRNAs were found in breast cancer (FDR < 0.05, |logFC|> 1.5), including 129 that were up-regulated and 216 that were down-regulated (Fig. 2A). Using Pearson correlation analysis, 132 lncRNAs significantly associated with disulfidptosis were identified (*p*.adj < 0.05, |R|> 0.15). Preprocessing, which includes feature selection, is crucial in machine learning, as is well known. It can somewhat increase the model's prediction accuracy in addition to reducing the complexity of the training model^49^. As a result, we prioritize the significance of characteristics using the random forest algorithm, screen out features that are closely related to the model, and improve the accuracy of the breast cancer prediction model. Four lncRNAs with the highest relative importance values and relative importance greater than 40 were selected as key factors for constructing a model to predict breast cancer subtypes (Fig. 2B,C).

We identified biomarkers by evaluating the predictive models (Figs. 2D–F). The expression levels of the four key lncRNAs used to build the model were significantly different among the five subtypes. In the Basal subtype, LINC00511 and LINC02188 had the highest expression (0.75–1 quantile, Q4), while LINC01488 had the lowest (0.25–0.5 quantile, Q2). LINC00511 was significantly higher than adjacent tissues (*P* < 0.001). In the Her2 subtype, GATA3-AS1 had the highest expression (Q4), while LINC01488 had the lowest (Q2). LINC00511 and LINC01488 were significantly higher than adjacent tissues (*P* < 0.001). In LumA and LumB subtypes, GATA3-AS1 had the highest expression (Q4), while LINC00511 had the lowest (0.5–0.75 quantile, Q3). LINC00511 and LINC01488 were significantly higher than adjacent tissues (*P* < 0.001), while LINC02188 was significantly lower (*P* < 0.001). In the Normal subtype, GATA3-AS1 had the highest expression (Q4), and LINC00511 and LINC01488 were significantly higher than adjacent tissues (*P* < 0.001).

### Evaluation of a model of disulfidptosis-associated lncRNAs for predicting breast cancer subtypes

The Support Vector Machine model (SVM) is a supervised learning algorithm utilized for data analysis via classification and regression^50^. The K-Nearest Neighbor (KNN) algorithm, on the other hand, is a straightforward instance-based learning technique^51^. Meanwhile, the Naive Bayesian (NB) classifier stands as a well-established supervised algorithm within the field of machine learning^52^. Subsequently, we harnessed Random Forest (RF) to construct a breast cancer subtype prediction model, incorporating these four pivotal algorithms for comparison: RF, KNN, SVM, and NB.

**Figure 3:**
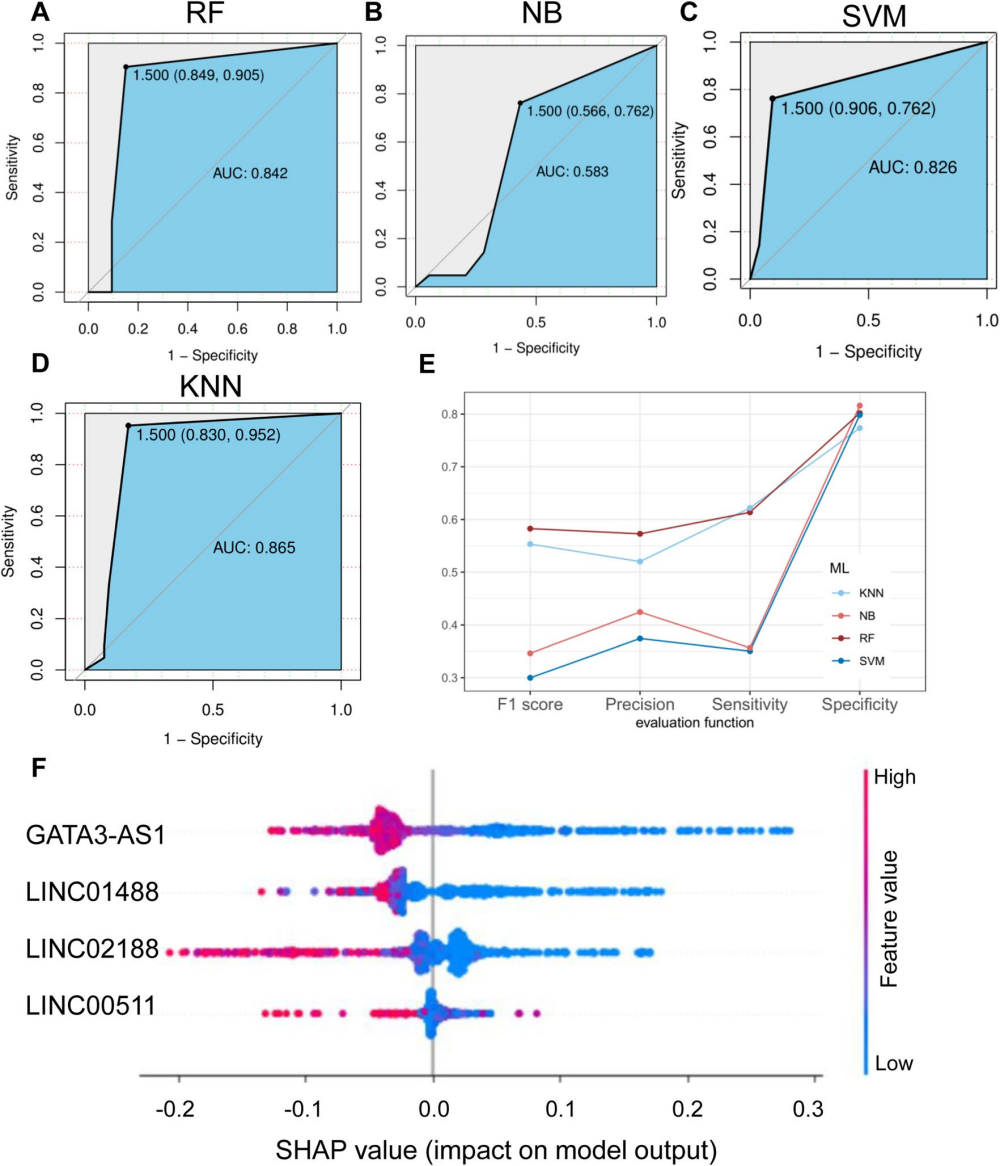
Evaluation of machine learning Model. (**A**) ROC curve of verification set (Random Forest model). (**B**) ROC curve of verification set (Naive Bayesian model). (**C**) ROC curve of verification set (Support Vector Machine model). (**D**) ROC curve of verification set (K-Nearest Neighbor model). The ROC curve was constructed through “pROC” R package (version:1.18.0, https://cran.r-project.org/web/packages/pROC/index.html). (**E**) Sensitivity (Sn), Specificity (Sp), Precision, and F1 Score of verification set. Line graphs was constructed through “ggplot2” R package. (**F**) The SHAP disease risk factor model identifies critical features and their impact ranges across the dataset. Color coding reflects feature values, with red denoting high values and blue indicating low values.

In Fig. 3A–D, we present the AUC (Area Under the Curve) results for these four machine learning models. RF achieves an AUC of 0.842, while NB records an AUC of 0.583, SVM achieves 0.826, and KNN attains 0.865. Figure 3E exhibits additional metrics, including Specificity (Sp), Sensitivity (Sn), Precision, and F1 score for the three machine learning models. For RF, Sp is 0.8, Sn is 0.6, Precision is 0.57, and F1 score is 0.58. KNN yields Sp of 0.77, Sn of 0.62, Precision of 0.52, and F1 score of 0.55. Meanwhile, NB displays Sp of 0.8, Sn of 0.36, Precision of 0.42, and F1 score of 0.35. SVM produces Sp of 0.8, Sn of 0.35, Precision of 0.37, and F1 score of 0.3.

While KNN demonstrates a higher AUC value than RF, it’s essential to consider that RF outperforms KNN in terms of Sp, Precision, and F1 score. Furthermore, the overall evaluation indices of RF surpass those of SVM and NB models, underscoring the robustness of the breast cancer prediction model constructed using Random Forest.

We then used the SHAP model to evaluate the role of key lncRNAs in our model. In the model, GATA3-AS1 had the most significant impact, while LINC00511 had the least. The results indicated that each key gene had a different contribution to the model (Fig. 3F).

### Immune infiltration and the disulfidptosis-associated lncRNAs

**Figure 4:**
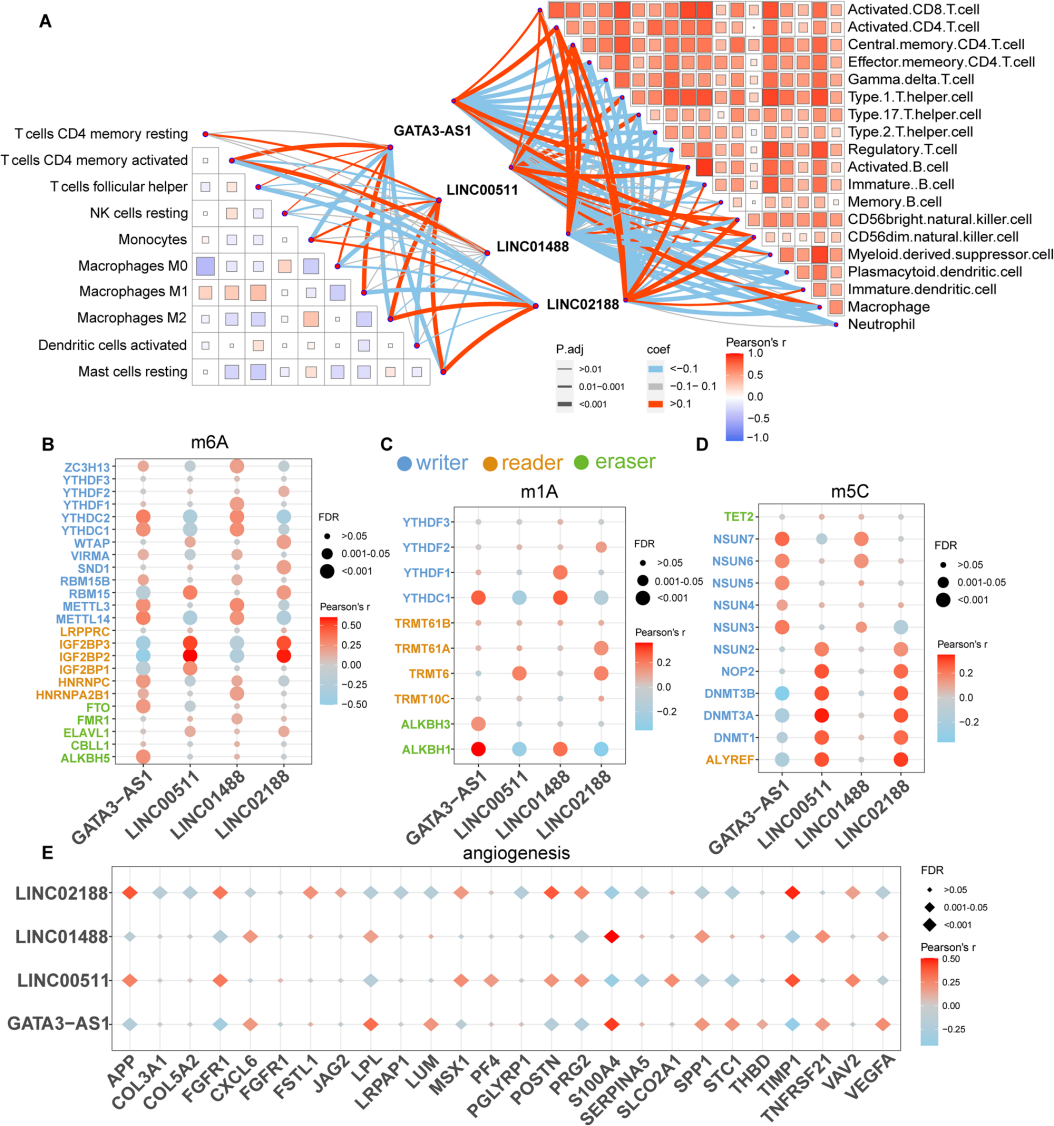
Key lncRNAs in breast cancer subtypes associated with immune infiltration, RNA methylation modification, and angiogenesis. (**A**) On the right, ssGSEA is used to assess the enrichment scores of immune pathways in breast cancer patients. Correlations exist between immune pathways and between key lncRNAs and immune pathways. On the left, CIBERSORT is used to assess the proportion of immune cells in breast cancer patients. Correlations exist between immune cells and between key lncRNAs and immune cells. Pearson’s r represents the correlation coefficient between immune cells, while coef represents the correlation coefficient between key lncRNAs and immune cells. *P*.adj represents the adjusted significance *P* value of key lncRNAs in breast cancer subtypes and immune cells. Correlation heatmap was constructed through “linkET” R package (version:0.0.4, https://rdrr.io/github/Hy4m/linkET/). (**B**)–(**E**) Key lncRNAs show correlation with m6A modification-related genes (**B**), m1A modification-related genes (**C**), m5C modification-related genes (**D**), and angiogenesis-related genes (**E**), respectively. The corrected *P* value (*P*.adj) was calculated using Bonferroni’s correction. Correlation heatmap was constructed through “ggplot2” package in R.

We conducted an immune infiltration assay to explore the relationship between disulfidptosis and immune infiltration in different breast cancer subtypes. CIBERSORT and ssGSEA were used to evaluate the immune infiltration of patients, and the correlation between key lncRNAs and immune cells was calculated. Key lncRNAs were found to have varying degrees of correlation with the level of immune cell infiltration (Fig. 4A).

### RNA methylation genes and the disulfidptosis-associated lncRNAs

We explored the relationship between disulfidptosis-related lncRNAs and RNA methylation in different breast cancer subtypes by assessing the correlation of key lncRNAs with RNA methylation genes. We observed diversity in the association between four lncRNAs and RNA m6A modifying genes (Fig. 4B). GATA3-AS1 and LINC01488 showed positive correlations with RNA m1A modifier gene expression, while LINC00511 showed a negative correlation. The correlation between LINC02188 and RNA m1A modifier genes varied (Fig. 4C). LINC00511, LINC01488, and LINC02188 were positively correlated with RNA m5C modification genes (Fig. 4D), while the association between GATA3-AS1 and m5C modifier genes was diverse (Fig. 4D).

### Angiogenic genes and the disulfidptosis-associated lncRNAs

Angiogenesis, the formation of new blood vessels, has been shown to be integral to cancer development^53^. Our study assessed the correlation of key lncRNAs with angiogenic genes to explore the relationship between disulfidptosis and angiogenesis in different breast cancer subtypes. Our results indicate that the relationship between the four lncRNAs and angiogenesis is intricate. Each of the four lncRNAs exhibited positive or negative correlations with multiple angiogenic genes (Fig. 4E).

## Discussion

Disulfidptosis is a recently discovered type of cell death that differs from apoptosis, autophagy, and ferroptosis. In this study, we established a prediction model for breast cancer subtypes based on 4 lncRNAs related to disulfidptosis. The model includes 3 lncRNAs that are highly expressed in breast cancer (GATA3-AS1, LINC00511, and LINC01488) and 1 lncRNA that is lowly expressed (LINC02188). LINC01488 and LINC00511 showed higher expression in the Basal subtype, while GATA3-AS1 showed higher expression in the Her2 and Normal subtypes. GATA3-AS1 and LINC02188 showed higher expression in the LumA and LumB subtypes.

In this study, LINC02188 was found to be associated with a reduced risk of Her2, LumA, and LumB breast cancer subtypes for the first time. Located on chromosome 16 and 658 bp in length, LINC02188 has been shown to be associated with the activation of various immune cells, RNA methylation modifier genes, and the expression of multiple angiogenic factors. The COPS3 protein is a subunit of the COP9 signalosome (CSN) that exerts deubiquitination and protein kinase activity in various processes^54^. PD-L1 is a ligand for programmed cell death protein 1 (PD-1) that inhibits T cell signaling by interacting with PD-1^55^. GATA3 is a transcription factor that plays an important role in the differentiation of mammary epithelium, urothelium, and T lymphocyte subsets^56^. GATA3-AS1 induces PD-L5 deubiquitination through the miR-1-676p/COPS3 axis while destabilizing GATA3 protein by promoting its ubiquitination, thus promoting TNBC progression and immune escape^57^. MMP13 is a matrix metalloproteinase that remodels the extracellular matrix and promotes cancer cell invasiveness^58^. LINC00511 promotes breast cancer proliferation, migration, and invasion through the miR-150/MMP13 axis. In HCC, LINC01488 inhibits metastasis and tumorigenesis via the miR-124-3p|miR-138-5p/vimentin axis^59^. Our research shows that LINC01488 is lowly expressed in the LumB subtype of breast cancer but highly expressed in the Basal, Normal, Her2, and LumA subtypes. We speculate that LINC01488 expression may be tissue-specific.

Plasmacytoid dendritic cells (pDCs) can recognize viruses and tumor cells and enhance the function of natural killer cells (NK cells), T cells, B cells, and other dendritic cells to promote cellular innate and adaptive immune responses^60^. T cells are lymphocytes that can kill tumor cells by recognizing tumor-specific or tumor-associated antigens, exerting anti-tumor immune effects and playing a key role in tumor monitoring^61,62^. Macrophages are important cells in the tumor microenvironment that can polarize into M1 or M2 phenotypes in response to different stimuli and signals^63^. M1 macrophages mainly induce the production of pro-inflammatory cytokines such as TNF-α, IL-1β, IL-6, and IL-12, which are conducive to anti-tumor effects^64^. In the Basal subtype, high expression of LINC02188 and LINC00511 may increase immune infiltration of tumor tissue by activating various immune cells, thereby inhibiting tumor development. Mast cells can promote tumor cell proliferation and invasion^65^. M2 macrophages secrete anti-inflammatory cytokines such as IL-10, CCL18, and CCL22, which are beneficial to cancer cell growth^66^. In the LumA and LumB subtypes, high expression of LINC01488 and GATA3-AS1 may activate Mast cells and M2 macrophages, promoting tumor immune escape and development.

RNA methylation plays a critical role in cancer development^67^. m6A is the most prevalent internal mRNA modification in eukaryotic cells and regulates multiple RNA processing steps^68^. The relationship between the four lncRNAs and m6A modifying genes is complex and has both positive and negative correlations. RNA m1A modification disrupts base pairing and can affect local RNA structure or protein-RNA interaction^69^. GATA3-AS1 and LINC01488 were positively correlated with RNA m1A modifier gene expression, while LINC00511 was negatively correlated. RNA m5C modification can promote mRNA nucleoplasmic transport, DNA damage repair, enhance mRNA stability and regulate mRNA splicing^70^. LINC00511, LINC01488, LINC02188 were all positively correlated with RNA m5C modification genes. Since DNMT3A, DNMT3B, and DNMT1 are also responsible for DNA m5C methylation modification, this suggests that LINC00511 and LINC02188 may promote DNA methylation while GATA3-AS1 reduces it.

This study has some limitations. Firstly, we only used the TCGA internal dataset for analysis and lack external validation. In the future, we need to verify the accuracy of the model using more clinical samples. Secondly, as disulfidptosis research is a novel and fast-growing field, more regulators may be discovered in the future and the model can be further optimized after a deeper understanding of the biological process of disulfidptosis. Additionally, this study was based on RNA profiling and cannot explain the direct molecular mechanism of disulfidptosis-related lncRNAs in breast cancer development at the protein level.

Prior research endeavors have explored the application of computational methodologies, notably machine learning models, in the realms of breast cancer diagnosis and prognosis. For instance, one study leveraged support vector machine (SVM) techniques, employing FTIR spectra from plasma, to detect breast cancer^71^. Another notable contribution by Mariia V. Guryleva et al. combined the Boruta algorithm with a Random Forest (RF) model to delineate genes associated with Polyunsaturated Fatty Acid (PUFA) metabolism changes in breast cancer, thereby facilitating breast cancer subtype prediction^72^. Hang et al. introduced an MRI-based multiparameter radiomics model that adeptly forecasts molecular subtypes and androgen receptor expression in breast cancer^73^. Additionally, Zheng et al. harnessed deep learning radiomics for the prediction of axillary lymph node status in early breast cancer^74^.

In contrast to prior studies, our research represents a pioneering effort in incorporating disulfidptosis-associated long non-coding RNAs (lncRNAs) into the signature screening process. This novel approach enabled the identification of four distinctive biomarkers showcasing differential expression patterns across various breast cancer subtypes. Remarkably, our findings also suggest an intriguing link between breast cancer subtypes and disulfidptosis phenomena.

Furthermore, it's noteworthy that previous investigations have predominantly focused on utilizing machine learning models trained on breast cancer images for tumor classification into neoplastic and benign categories. While these studies exhibit higher accuracy and specificity, it's imperative to acknowledge the resource-intensive nature of training such models using expensive and time-consuming breast cancer images.

## Conclusions

Here, we constructed a breast cancer subtype prediction model containing 4 key lncRNAs using a random forest model. We found strong correlations between key lncRNAs in the model and immune milieu, RNA methylation, and angiogenesis. Our findings reveal the potential function of disulfidptosis-asscociated lncRNAs in breast cancer subtypes and provide a new direction for improving individualized treatment of breast cancer.

### Supplementary Information

Supplementary Table S1.

## Data Availability

All relevant data are within the paper and its Supporting Information files. The data that support the findings of this study are openly available in TCGA database at https://portal.gdc.cancer.gov/.

## Abbreviations

LumA: Luminal A
LumB: Luminal B
Her2: HER2-enriched
Basal: Basal-like
Normal: Normal-like
H2S: Hydrogen sulfide
RSS: Reactive sulfur species
lncRNAs: Long noncoding RNAs
FDR: False discovery rate
RF: Random forest
VIMP: Variable importance
ssGSEA: Single-sample gene set enrichment analysis
AAGs: Angiogenesis-associated genes
*P*.adj: Corrected *P* value
AUC: Area under the curve
ROC: Receiver operating characteristic
Sp: Specificity
Se: Sensitivity
NB: Naive Bayesian mode
SVM: Support vector machine
KNN: K-nearest neighbor
CSN: COP9 signalosome
PD-1: Programmed cell death protein 1
pDCs: Plasmacytoid dendritic cells
NK cells: Natural killer cells
